# Plant- and Animal-Based Antioxidants’ Structure, Efficacy, Mechanisms, and Applications: A Review

**DOI:** 10.3390/antiox11051025

**Published:** 2022-05-23

**Authors:** Edirisinghe Dewage Nalaka Sandun Abeyrathne, Kichang Nam, Xi Huang, Dong Uk Ahn

**Affiliations:** 1 Department of Animal Science, Uva Wellassa University, Badulla 90000, Sri Lanka; sandun@uwu.ac.lk; 2 Department of Animal Science & Technology, Suncheon National University, Suncheon 57922, Korea; kichang@scnu.ac.kr; 3 Key Laboratory of Environment Correlative Dietology, Ministry of Education, National Research and Development Center for Egg Processing, College of Food Science and Technology, Huazhong Agricultural University, Wuhan 430070, China; huangxi@hzau.edu.cn; 4 Department of Animal Science, Iowa State University, Ames, IA 50011, USA

**Keywords:** animal-based antioxidants, plant-based antioxidants, mechanism, function, application

## Abstract

Antioxidants are compounds that normally prevent lipid and protein oxidation. They play a major role in preventing many adverse conditions in the human body, including inflammation and cancer. Synthetic antioxidants are widely used in the food industry to prevent the production of adverse compounds that harm humans. However, plant- and animal-based antioxidants are more appealing to consumers than synthetic antioxidants. Plant-based antioxidants are mainly phenolic compounds, carotenoids, and vitamins, while animal-based antioxidants are mainly whole protein or the peptides of meat, fish, egg, milk, and plant proteins. Plant-based antioxidants mainly consist of aromatic rings, while animal-based antioxidants mainly consist of amino acids. The phenolic compounds and peptides act differently in preventing oxidation and can be used in the food and pharmaceutical industries. Therefore, compared with animal-based antioxidants, plant-based compounds are more practical in the food industry. Even though plant-based antioxidant compounds are good sources of antioxidants, animal-based peptides (individual peptides) cannot be considered antioxidant compounds to add to food. However, they can be considered an ingredient that will enhance the antioxidant capacity. This review mainly compares plant- and animal-based antioxidants’ structure, efficacy, mechanisms, and applications.

## 1. Introduction

Many modern non-communicable diseases are related to oxidative stress, mainly generated by the imbalance between the formation and neutralization of the prooxidants [1]. Food proteins are good sources of antioxidants [2]. Antioxidants are natural or synthetic compounds that inhibit or delay the oxidation process at relatively low concentrations. They can be divided into primary and secondary compounds [3,4,5]. Various plants and vegetables are good sources of natural antioxidants such as vitamin C, carotenoids, anthocyanins, and phenols [4,6,7,8]. Antioxidants can interact with free radicals before damaging the host cells. The function of antioxidants can be explained in several mechanisms: scavenging free radicals that initiate and propagate peroxidation, chelating metal ions that accelerate the oxidation process, quenching ^●^O_2_^−^ ions to prevent the formation of hydroxyl radical, breaking the auto-oxidation chain reaction, and lowering the localized O_2_ concentration in the product [7]. These antioxidants are considered safe products to add to any food product at the industrial level [9].

In the process, maintaining the nutritional value of the basic foods is very important. However, most food components containing fat and oils are subjected to oxidation during processing and storage. Lipid oxidation in processed food is one of the most common problems humans face. Various approaches have been taken to reduce oxidative stress and aging of the cells; adding antioxidants to foods is one of the main methods [7,10].

Phenolic compounds from plants can protect humans against cancer and many other diseases, including cardiovascular and inflammatory disorders. Therefore, adding the phenolic compounds to the food system is widely used to prevent various human diseases [1,8,11]. Vitamin C is a common natural antioxidant that reduces the serum uric acid level, which causes gout, and reduces the risk of having a stroke, and chronic and degenerative diseases. Vitamin E (tocopherol) is another antioxidant that protects the cell membrane, prevents lipid peroxidation, and chelates reactive oxygen species (ROS). Carotenoids, lutein, zeaxanthin, and lycopene reduce cancer and metabolic diseases. In addition to antioxidants, flavones also act as anti-inflammatory agents [7].

These natural antioxidants are used in many indigenous medicines and traditional foods. Natural antioxidants are widely used to prevent oxidative stress in meat and meat products. These natural compounds prevent lipid oxidation in meat products and improve the product’s physicochemical, chemical, and sensory properties [9]. Plants seeds rich in polyphenols have been used as natural antioxidants for a long time. The phenolic compounds are present in the coat of the seeds in many fruits, cereals, and legumes [10]. These natural antioxidants are good replacements for the synthetic antioxidants used in the food industry. Natural antioxidant compounds, such as anthocyanin, improve the color of the meat product while they are responsible for the oxidative stability in the meat product [9]. Furthermore, these natural antioxidant compounds can improve the sensory properties of meat and meat products. They are used to reduce the off-odor and off-flavor while improving the color of the processed meat [12]. These antioxidant compounds are used as anticancer agents with low toxicity and specificity and high availability. Combining these compounds with food components can have cytoprotective effects on normal cells and cytotoxic effects on cancer cells [10]. Many plant-based products are in traditional Chinese, Islamic, and ancient Rome medicines and the herbal teas they drink. Plant products taken from pine trees, including the bark, shoots, and resins, treat urinary tract diseases, digestive tract disorders, and problems associated with the nerve system, respiratory system, and skin problems [13].

A high level of reactive oxygen species (ROS) produced in the muscle tissues can increase the release of stress hormones into the bloodstream which stimulate the release of enzymes, cortisol, and catecholamines. Oxidation can lead to the formation of off-flavors and nutrient losses while reducing the shelf life of the products. Antioxidants are important to reduce oxidative stress in live animals and prevent the development of the off-flavor and rancid odor, discoloration, and accumulation of toxic compounds in meat during processing and storage.

Providing antioxidants to animals before transporting them to the slaughterhouse showed better quality meat production since it reduced the stress during loading and unloading and transportation even with poor road conditions [12]. Natural antioxidants can be used as antimicrobial agents to control foodborne pathogens such as *Salmonella*. These compounds can be used in several meat-based products, especially beef, poultry, and pork, to control foodborne pathogens [14]. Therefore, this review aims to compare the plant-based and animal-based antioxidants, their structure, and potential use in the food industry.

## 2. Antioxidants from Plant Sources

Plants contain many natural compounds that have antioxidant activity. These compounds can be categorized into vitamins (Vitamin C and E), polyphenols (flavonoids, phenolic acids, stilbenes, lignans), and terpenoid groups. Fruits and vegetables are rich sources of vitamin C and E. Among the fruits are the family Rosaceae (sour cherry, strawberry, blackberry), Empetraceae (cowberry), Ticaceae (blueberry), Asteraceae (sunflower seed), and Punicaceae (pomegranate), which are rich sources of these vitamins. Broccoli, brussels sprouts, green cabbage, tomatoes, cauliflowers, lettuce, and leeks are vegetable groups with high vitamin C and E. Vitamins in plants act as primary antioxidant substances [15]: Vitamin E acts as an essential lipid-soluble antioxidant, while vitamin C protects against oxidative stress-induced cellular damages [16,17]. Both vitamin E and vitamin C are used as antioxidants in foods, but the effect of vitamin C is marginal.

**Figure 1:**
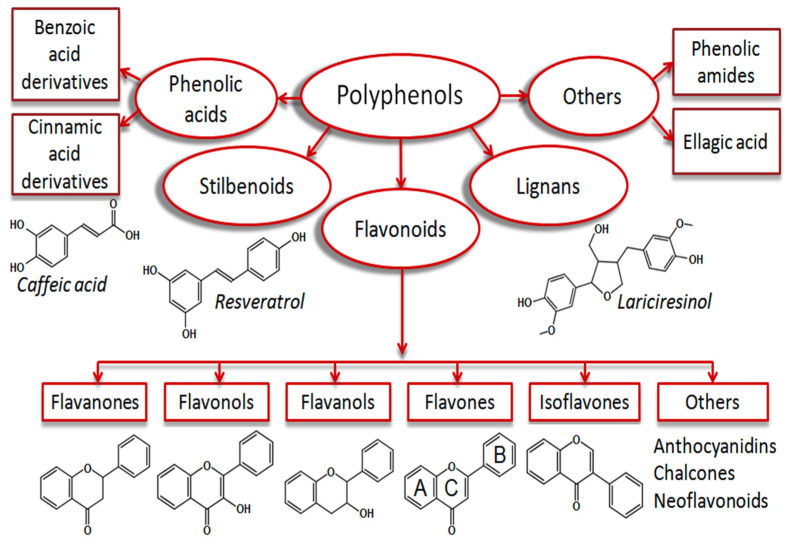
Plant phenolic compound breakdown. This Figure is reproduced from ref. [23]. Copyright 2019 (Dirimanov & Högger, 2019).

Polyphenols are the major plant antioxidants with various structural and functional characteristics and biological properties [11,18,19]. Phenolic compounds synthesize from phenylalanine or tyrosine through the shikimic acid pathway. They can vary from simple compounds to conjugated complex substances. These compounds vary from 500 to 4000 Da molecular weight, and over 12 phenolic hydroxyl groups are found among the phenolic compounds [4,20]. They can be subcategorized into phenolic acids, flavonoids, stilbenes, and lignans [6,21]. They are found in plant foods such as fruits, cereals, seeds, berries, and plant-based products such as wine, tea, and vegetable oils [22,23]. The summary of the breakdown of the phenolic acids is shown in Figure 1.

**Figure 2:**
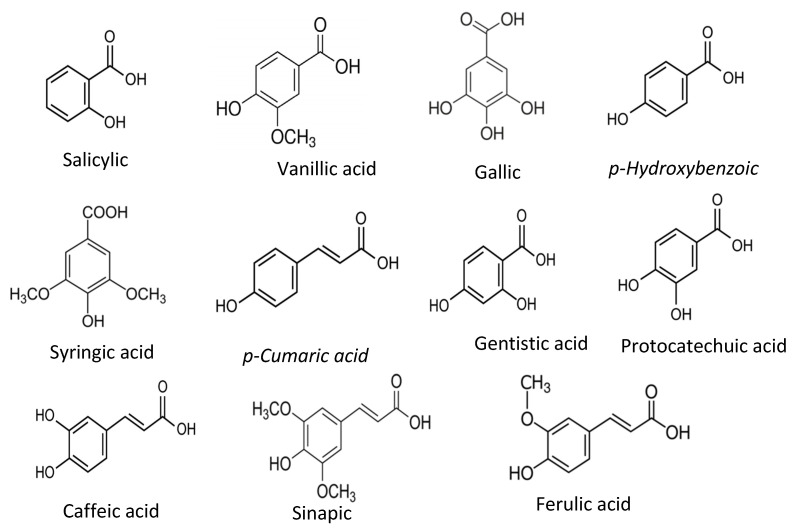
Common phenolic acids found in food plants.

Phenolic acids are the derivatives of benzoic acids and cinnamic acids, and salicylic acid, gentisic acid, *p*-Hydoxybenzoic acid, protocatechuic acid, vanillic acid, syringic acid, gallic acid, *p*-coumaric acid, ferulic acid, caffeic acid, and sinapic acid are among the most common phenolic compounds present in the food plants and common phenolic acid compounds illustrated in Figure 2.

The antioxidant activities of those phenolic acids are confirmed by several antioxidant assays, including the DPPH radical scavenging assay, ABTS radical scavenging activity, and β-carotene assay [5]. The radical scavenging activities of these phenolic acids mainly depend on the hydroxy moieties attached to the phenyl rings of benzoic and cinnamic acids [24,25].

**Figure 3:**
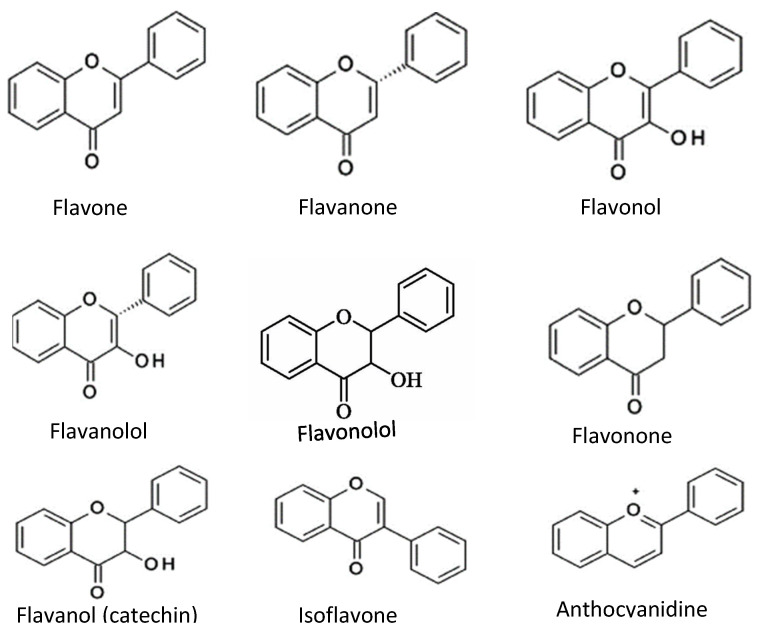
Chemical structures of common flavonoids.

Flavonoids consist of two outer aromatic rings with three carbon rings. These flavonoids include flavone, flavanol, flavanone, flavanonol, flavonone, flavononol, flavanol (catechin), isoflavone, and anthocyanidin (Figure 3) [6]. The derivatives of the flavonoids have inhibitory properties against acetylcholinesterase [26].

**Figure 4:**
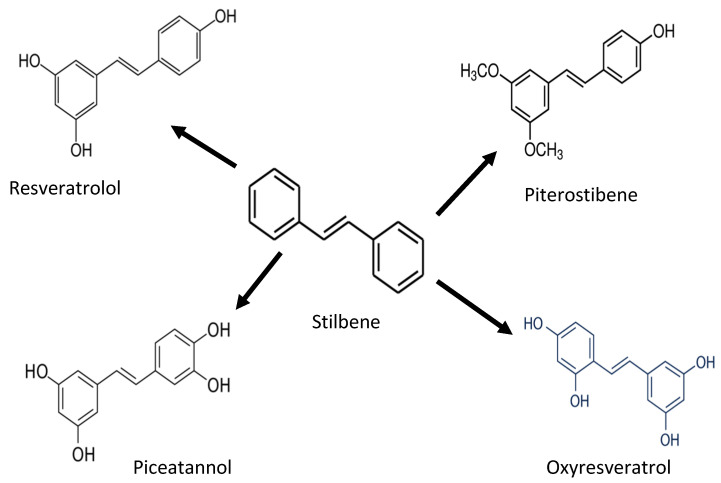
Chemical structure of stilbene and its derivatives.

Stilbenoides/stilbenes are another phenolic compound commonly found in berries, grapevines, and peanuts. Stilbenoids are hydroxylated stilbene derivatives (i.e., resveratrol). The most common stilbenes are piceid, resveratrol, piceatannol, and pterostilbene (Figure 4). However, only resveratrol has antioxidant potential against proteins and lipids [27,28,29]. Endogenous compounds such as superoxide dismutase (SOD), catalase, and glutathione neutralize oxidative stress induced by UV radiation. Stilbenes, such as resveratrol, can increase the activity of antioxidant enzymes (glutathione *S*-transferase) and increase the SOD level. In addition, pterostilbenes reduce oxidative damage by activating the endogenous antioxidant enzymes [29].

**Figure 5:**
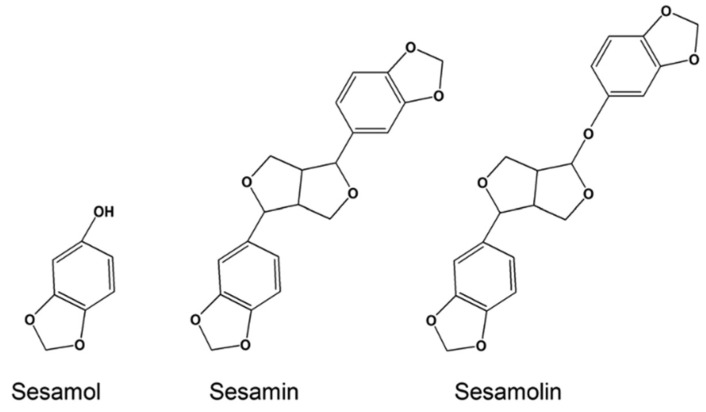
Structure of sesamol, sesamin, and sesamolin. This Figure is reproduced from ref. [34]. Copyright 2016 Wiley.

Lignans are precursors to phytoestrogens and synthesized from phenylalanine with dimerization of substituted cinnamic alcohols. Sesame seeds and flax seeds are the main sources of lignans. Secoisolariciresinol, matairesinol, pinoresinol, and lariciresinol are the common lignans from flax seeds and sesamin, sesamoiln, sesamolinol, and sesaminol are the common lignans from sesame seeds [30]. Sesamin and sesamolin possess antioxidant, neuroprotective, and anticancer activities, but sesamol, their decomposition product during the roasting process of sesame seeds (Figure 5), is the major antioxidant of the sesame seeds [31,32,33,34]. The common plant-based phenolic antioxidants are summarized in Table 1.

**Figure 6:**
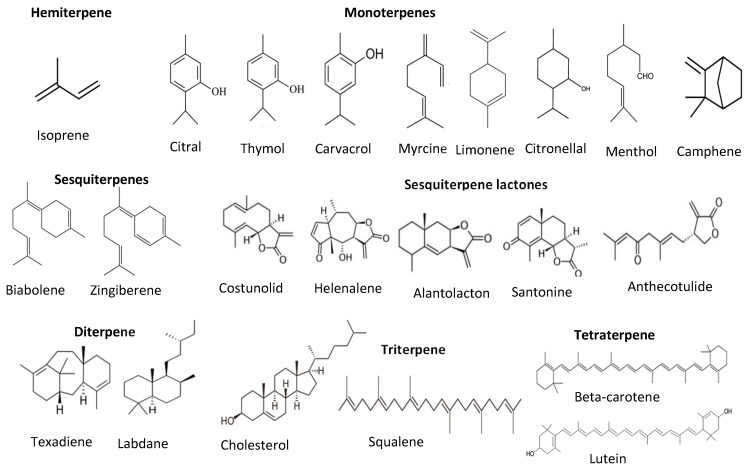
Structure of different classes of terpenoids.

Terpenes and terpenoids are also good antioxidants from plant sources. They are the largest secondary metabolites of plants. Terpenes and terpenoids contain a hydrocarbon skeleton with five carbons (isoprene), and two or more isoprene molecules polymerize and form various terpenes. Most of them are non-polar compounds [45]. Plant oils such as pine oil, vegetables such as carrots, and some fruits such as lemon and orange are rich sources of terpenes and terpenoids. These compounds can further classify into monoterpenes (C-10), sesquiterpenes (C-15), diterpenes (C-20), triterpenes (C-30), tetraterpenes (C-40), or carotenoids, polyterpenes, norisopernoids, and sesquatreterpenes (Figure 6). These compounds have antioxidant and antimicrobial activities, contributing odor and flavor and other health-promoting properties such as relieving stress and depression, reducing depression and migraines, and antiaging and anticancer properties [35,46].

**Figure 7:**
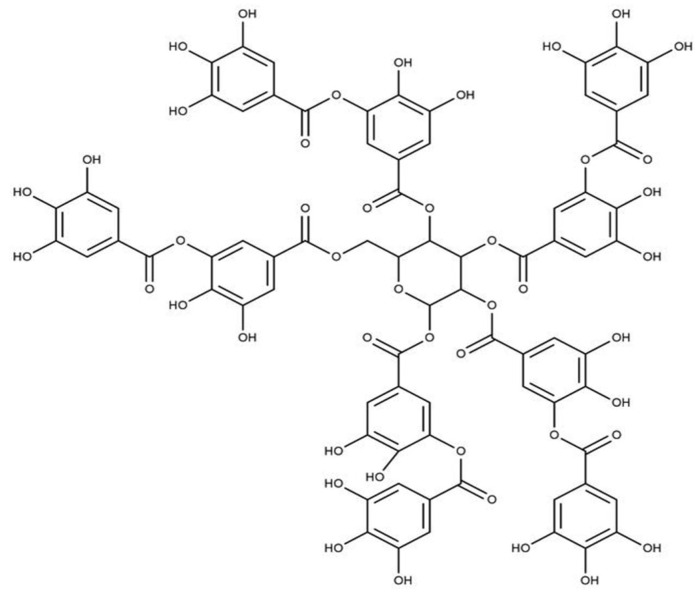
Structure of Tannic acid.

Tannins are another group of phenolic antioxidants in plants and can be divided into two main sub-classes: condensed tannins and hydrolyzable tannins. The condensed tannins are biopolymers based on flavan-3-ols, and gallic and ellagic acid derivatives (gallotannins and ellagitannins) are the main components with antioxidant properties [36,39]. Gallotannins are natural polymers formed by the esterification of D-glucose and gallic acid hydroxyl groups. Proanthocyanidins can donate hydrogen atoms/electrons and act as an antioxidant compound. Proanthocyanidins are abundant in green tea and bearberry [24,38]. Tannin extracts from red beans, adzuki beans, lentils, fava beans, and broad beans showed better antioxidant activity than the flavonoids and phenolic acids separated from the same plant materials [37,40,41]. The chemical structure of the tannic acid is shown in Figure 7.

Oilseeds, cereal grains, legumes, tea, coffee, tree nuts, fruits, and berries are excellent sources of plant antioxidants. Among the oilseeds, rapeseed and canola seeds have very high levels of phenolic compounds [24,47]. Sinapine (choline esters of sinapic acid) and sinapic acids are the main phenolic compounds in rapeseed [48]. Most cereals contain phenolic compounds, and ferulic acid is the most dominant type. However, some wheat cultivars contain high levels of *p*-coumaric, sinapic, and caffeic acids. Rice and rice bran contain γ-oryzanol, an ester of triterpene alcohols, and plant sterols [42] with strong antioxidant properties. Legumes also contain high phenolic acid, flavonoids, and tannins. Catechin, epicatechin glucosides, procyanidin dimers, quercetin glucoside, and *p*-coumaric are the main phenolic compounds in green lentils [24]. The leaves and seeds of legumes are considered a good source of lignans. Lamiaceae is a good source of phenolic acid, rosmarininc and caffeic acid, flavonoids, hispidulin, nepetin, luteolin, and apigenin [43]. Oregano (*Origanum vulgare* L.) is another plant that belongs to the family and is rich in phenolic antioxidant compounds such as rosmarinic acid and chlorogenic acid and flavonoids such as hyperoside and isoquercitrin [44].

**Figure 8:**
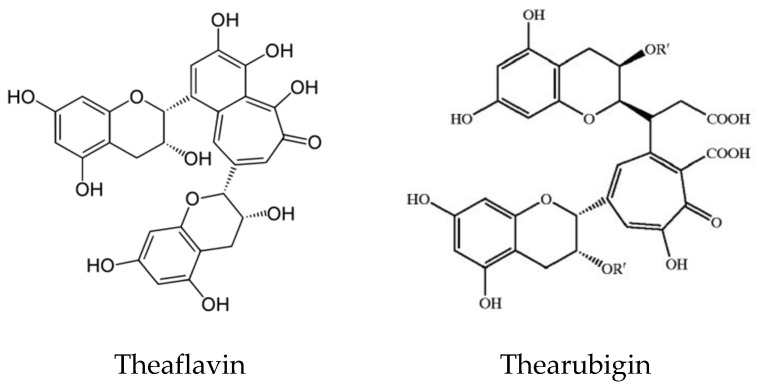
Structure of Theaflavin and Thearubigin in black tea.

**Figure 9:**
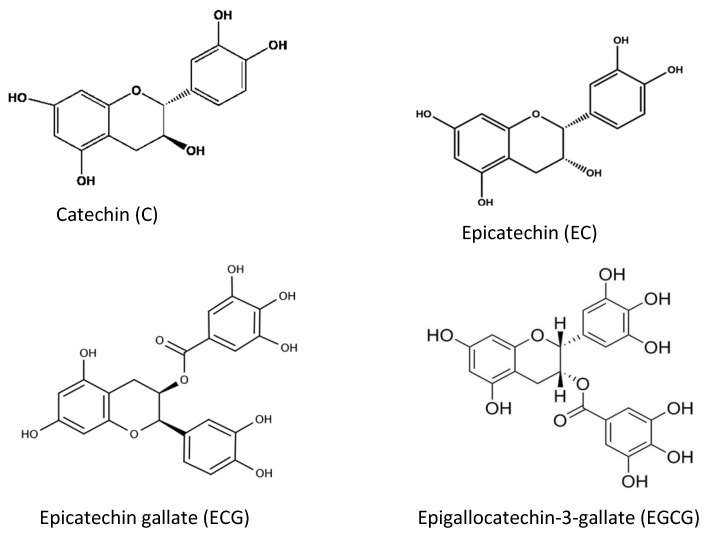
Different chemical structures of catechins found in green tea.

Tea and coffee are also rich sources of natural antioxidant compounds. Different antioxidants are found in green tea and black tea: theaflavin and thearubigin are the main antioxidant compounds in black tea (Figure 8), while the isomers of catechins are the main active antioxidant compounds in green tea (Figure 9). These are polyphenols, and the content varies among cultivars and by the method of processing [49,50,51].

Chlorogenic acids and their derivatives, including caffeoylquinic acids, caffeoylquinic acids, feruloyquinic acids, *p*-coumaroylquinic acids, caffeic acids, and ferulic acids, are the predominant phenolic compounds in coffee beans [52]. However, during heat processing, these phenolic compounds convert to quinolones and melanoidins [53]. Tree nuts are also rich sources of phenolic compounds: catechin, epicatechin, epicatechin 3-gallate, and procyanidins are rich in hazelnut kernels [54], and chlorogenic acid, caftaric acid, ferulic acid, gentisic acid, caffeic acid, *p*-coumaric acid, sinapic acid, isoquercitrin, rutozid, myricetin, fisetin, quercitrin, quercetin, luteolin, kaempferol, patuletin, hyperoside, and apigenin are rich in walnuts [55].

Fruits such as apples contain high amounts of polyphenols, hydroxycinnamates, flavonols, anthocyanins, and dihydrochalcones [56]. The main antioxidant compounds in plums are gallic acid, rutin, resorcinol, chlorogenic acid, catechin, and ellagic acid [8]. Berries, including strawberry, blackberry, blueberry, and cranberry, are rich in anthocyanins, flavonols, phenolic acids, and hydrolyzable tannins [57].

Phenolic compounds are mainly extracted using subcritical water. The extraction of phenolic compounds is based on hot water treatments called brewing. The temperature of the water and soaking duration determine the yield of the extracted phenolic compounds [53,58,59]. Solvent extraction is used to separate phenolic compounds from plant materials. Solvents such as methanol, acetone, ethanol, propanol, dimethylformamide, and ethyl acetate are mainly organic solvents. Different percentages of organic solvents separate the phenolic compounds [47]. The solvent and sample extraction time and ratio determine the yield and the purity of the phenolic compounds separated [60,61]. Furthermore, alkaline and acid hydrolysates separate these phenolic compounds [62].

The separated phenolic compounds incorporated into edible oils rich in unsaturated fatty acids effectively prevented lipid oxidation [63,64,65,66,67,68,69,70]. Combining the plant extracts rich in phenolic compounds, and synthetic antioxidants maintained the quality of many food products during storage [71]. Phenolic compounds are also widely used as natural antioxidants in meat and meat products. Among the plant extracts, almond seeds, grape seeds, and rosemary extracts are widely used as a natural preservative in beef and pork-based products [72,73,74]. Incorporating rosemary and green tea extracts into butter effectively prevented lipid oxidation [75]. Other plant extracts are also added to prevent lipid oxidation in seafood-based products [76,77].

## 3. Antioxidants from Animal Sources

### Antioxidant Animal Proteins and Peptides

Egg white proteins such as ovotransferrin and phosvitins are potential antioxidant proteins [78,79,80]. The main antioxidant mechanism of these proteins is preventing metal-catalyzed lipid oxidation by chelating ionic irons [81]. Ovotransferrin, especially, exhibits a distinct thiol-linked self-cleavage activity with binding with the metal ions [80].

Many peptides produced from proteins by enzymatic hydrolysis or other physicochemical treatments showed antioxidant activities. The antioxidant activity of these peptides depends on their amino acid sequence, composition, and length [79,82,83]. These antioxidant peptides are produced from the proteins of milk, fish, egg, meat, and the by-products (head, skin, fins, intestine, blood). Bioactive peptides are produced from various proteins and have specific physiological effects [84,85,86]. Many bioactive peptides are used in therapeutic medicine, mostly in traditional medicines. The bioactive peptides should be non-toxic and can be easily excreted and destroyed, and they produce using enzymatic hydrolysis. The physiological activities of bioactive peptides have been proven in both in vitro and in vivo studies [87,88,89]. Many bioactive peptides from animal sources are produced during processing, especially fermentation. During fermentation, proteins hydrolyze into polypeptides, and further microbial enzymes are broken down into smaller peptides. Many exopeptidase enzymes can act on the polypeptides’ N or C terminals and break down into smaller dipeptides, tripeptides, or even single amino acids, all of which can act as antioxidative bioactive compounds [88].

Milk and milk by-products, such as whey, are very good sources of proteins to produce bioactive peptides, and they were the first animal protein sources used to produce peptides with antioxidant activities. Milk contains two endogenous protease enzymes that break down milk proteins [90], and these enzymes produce peptides with an amino acid sequence of VLPVPQK that show antioxidant activity [88,91]. Sheep milk also contains several peptides that have antioxidant activities. Those peptides were the products of αs1-casein and αs2 casein by *Bacillus* spp. [92]. The enzymatic hydrolysis of camel milk with pepsin, papain, and alcalase produced peptides with the amino acid sequences of RLDGQGRPRVWLGR, TPDNIDIWLGGIAEPQVKR, and VAYSDDGENWTEYRDQGAVEGK, and they showed strong antioxidant activities [93]. The hydrolysis of buffalo milk casein with pepsin, trypsin, and chymotrypsin produced bioactive peptides with the amino acid sequence of VLPVPQK, which showed strong antioxidant activity. The peptides’ activity was evaluated using DPPH radical scavenging activity and Trolox equivalent antioxidant capacity (TEAC) [94]. Hydrolysis of bovine and ovine milk proteins produced peptides with antioxidant activities; a peptide with the amino acid sequence of YFYPEL was produced from the bovine casein with pepsin, and the hydrolysis of bovine κ-casein with pepsin, trypsin, and chymotrypsin produced antioxidative peptides [95]. Milk peptides with amino acid sequences of ARHPHPHLSFM, AVPYPQR, NPYVPR, and KVLPVPEK showed strong antioxidant activity and inhibited lipid peroxidation in Caco-2 cells activating the Keap1-Nrf2 system [96].

Various cheese products, such as Cheddar, Gouda, cottage cheese, Pategrás, and Crescenza cheese, contain bioactive peptides that bind metal ions and slow lipid oxidation [91,97]. Peptides with antioxidant activity are also found in the water-soluble extracts of yogurt. These antioxidative peptides prevented the formation of free radicals or scavenged free radicals and active oxygen species. Cheddar cheese produced from buffalo milk contained high amounts of bioactive peptides derived from α_s_- and β-casein, and these peptides promoted glutathione synthesis [85,90,97,98].

Meat and meat products contain bioactive peptides because they are rich in high-quality proteins [91,95]. Postmortem aging of meat can produce bioactive peptides with a molecular weight of less than 3 kDa, but the amount and type of peptides produced can differ depending on the pH, temperature, and enzymes (e.g., trypsin, chymotrypsin, elastase, pepsin, and carboxypeptidase) involved. Many antioxidant peptides were produced from beef, pork, mutton, chicken, deer, duck, and marine species, and their sizes are 2–20 amino acids long and were produced mainly from the myofibrilla and sarcoplasmic proteins [85,91,99,100,101]. Papain hydrolysis of pig proteins produced peptides with a molecular weight between 3 and 10 kDa, and the peptides with amino acid sequences of DAQEKLE, AKHPSDFGADAQ, and AKHPSDFGADAQA showed strong antioxidant activities [102]. Peptides produced from myofibrillar proteins with the amino acid sequences of KRQKYD, EKERERQ, KAPVA, PTPVT, RPR, GLSDGEWQ, GFHI, DFHING, and FHG also showed strong antioxidant activities [91]. Hydrolysis of porcine myofibrilla proteins with papain and actinase E produced five antioxidant peptides with the amino acid sequences of DSGVT, IEAEGE, EELDNALN, VPSIDDQEELM, and DAQEKLE [103].

Some peptides with strong antioxidant activities were identified in fermented and cured meat products; the peptides had the amino acid sequences of DSGVT, IEAEGE, EELDNALN, VPSIDDQEELM, DAQEKLE, ATA, SLTA, VT, SAGNPN, GLAGA, DLEE, FGG, and DM [103,104]. Peptides produced from traditional Chinese chicken products, with the amino acid sequences of HVTEE and PVPVEGV, also showed antioxidant activities [105]. Three oligopeptides with two to four amino acids (ALTA, SLTA, and VT) showed antioxidant properties and antioxidant activity in both in vitro and in vivo studies [104,106]. These antioxidative peptides are suggested as good alternatives to the synthetic antioxidants used in the food industry [87,101,107,108], but without practicality.

Not only meat and meat-based products but also the by-products produced during meat processing can be used to produce peptides that have antioxidant activity [84,86,88]. Skin collagen and blood proteins are commonly used to produce bioactive peptides among the by-products. Hydrolyzing pig skin collagen produces a mixture of antioxidant peptides with the amino acid sequences of QGAR, LQGM, LQGMH, and HC. Furthermore, in a trial carried out with water buffalo horn proteins, three antioxidant peptides having the amino acid sequences of QYDQGV, YEDCTDCHN, and AADNANELFPPN were produced [101,109]. Hydrolysis of broiler hen skin with elastase produced peptides with amino acid sequences of GAHTHPRLPFKPR, GMPGFDVR, and ADASVLPK, which showed strong antioxidant activity against DPPH and ABTS radical scavenging activity [110]. Collagen extracted from pigs hydrolyzed with the pancreas, papain, and protease produced peptides with strong antioxidant activities. Most of the peptides produced were dipeptides, and proline, glycine, and hydroxyproline were the dominant free amino acids [111]. Blood plasma hydrolysates (with Alcalase^R^ 2.4 L) also showed strong antioxidant activities; the peptides responsible for the antioxidant activity were GAHQPSG and QQPVRDOQ [112]. Alcalase, pepsin, trypsin, papain, and flavorzyme are the most used enzymes to produce antioxidative peptides from the slaughterhouse blood [113]. Porcine liver proteins hydrolyzed using alcalase, papain, and pepsin produced fifteen oligopeptides with strong antioxidant activities [114].

Poultry eggs are also considered one of the best sources of bioactive peptides production, and many of the peptides showed health benefits [78,83,91]. Pepsin, trypsin, chymotrypsin, and proteases produced peptides with antioxidant activities from egg proteins. Among the egg white proteins, ovalbumin, ovotransferrin, lysozyme, ovomucin, and ovomucoid are commonly used to produce peptides with antioxidant activity [79,83,115]. Peptides (WNIP, GWNI, IRW, and LKP) from ovotransferrin showed strong antioxidant activity, and the peptides from ovalbumin using pepsin and protease showed strong antioxidant activities [116]. In addition, peptides produced from other egg white proteins (ovomucin and ovomucoid) using heat treatment at a high pH or enzymatic hydrolysis showed strong antioxidant activities [79,80]. These peptides derived from egg white proteins showed significant metal- (Fe^2+^ and Cu^2+^) chelating activities and prevented oxidation. Another study showed that egg white proteins hydrolyzed with different enzymes produced peptides (VYLPR, YLGAK, GGLEPINFN, ESKPV, DVYSF, and DSTRTQ) with antioxidant activity [83].

Hydrolysis of yolk proteins with pepsin and pancreatin produced peptides with amino acid sequences of WYGPD and KLSDW with antioxidant activity such as synthetic antioxidants [78,115]. Furthermore, the peptides (LMSYMWSTSM, LELHKLRSSHWFSRR, and LELHKLRSSHWFSRR) that are produced from yolk phosvitin showed strong antioxidant activities [115]. Egg yolk peptides with the amino acid sequences of WYGPD, KLSDW, KGLWE, YINQMPQKSRE; YINQMPQKSREA, VTGRFAGHPAAQ, LMSYMWSTSM, LELHKLRSSHWFSRR, RASDPLLSV, RNDDLNYIQ, LAPSLPGKPKPD, AGTTCLFTPLALPYDYSH, QSLVSVPGMS, and YIEAVNKVSPRAGQF showed antioxidant activities, verified with several antioxidant assays, including the DPPH radical scavenging assay [83].

Di- and tripeptides such as carnosine, anserine, glutathione, and ophidine found in meat, poultry, and fish showed good antioxidant activities [3,100,101,106]. Carnosine, anserine, and ophidine are similar in their structure and capable of scavenging free radicals. They can inhibit lipid oxidation catalyzed by ionic iron, hydrogen peroxide-activated hemoglobin, singlet oxygen, and other free radicals [82,117]. Glutathione also acts as an antioxidant by neutralizing (reducing) reactive oxygen species and preventing oxidant-mediated cell death [118]. However, the amount of these compounds present in meat depends on breed, age, gender, and breeding program [101]. Some amino acid monomers such as glutamine (Q), asparagine (N), leucine (L), phenylalanine (F), isoleucine (I), methionine (M), valine (V), alanine (A), cysteine (C), and tyrosine (Y) also show antioxidant activities [83]. The animal-based antioxidant proteins and peptides are listed in Table 2.

## 4. Differences between the Antioxidants from Plant and Animal Sources

Most of the antioxidant compounds from plant sources are present in seeds, fruits, flowers, and leaves as active forms, while the animal-derived antioxidants are produced from proteins during digestion, or by hydrolyzing proteins using enzymes, and are functional only after the functional peptides are released from the proteins [72,91,113]. Some of the peptides and single amino acids have anti-inflammatory, hypoglycemic, antithrombotic, and ACE inhibitory activities, which are important for human health [88]. All the antioxidant compounds from plant sources (phenolic compounds, vitamins, and terpenoids) are produced as part of their metabolisms or tools for survival or defense against pests and diseases. These antioxidant compounds act as ROS scavengers in the plants, attractants for pollinations, protectants from insects or wounds, and improve metabolisms [44,72,91].

Different plant sources have different antioxidant compounds; most fruits and berries contain anthocyanins, catechins, flavanols, phenolic acids, and stilbenes. Tea and herbs are rich in tannins, catechins, and flavonoids [72,119]. The plant-derived antioxidant compounds are extracted using hot water, solvent, and alkaline water. On the other hand, enzymatic hydrolysis produces animal-based antioxidants after separating proteins from animal tissues. Pepsin, trypsin, elastase, chymotrypsin, proteases, and alcalase are some of the most common enzymes used to produce bioactive peptides with antioxidant activity. These bioactive peptides contain 3–20 amino acids [3,6,69,72,96,119] and are usually less than 6 kDa in molecular weight [82].

Plant-based natural antioxidants contain at least one phenyl ring with resonance double bonds and at least one OH- residue attached to the phenyl ring structure, whereas the animal protein-derived antioxidants (peptides) contain glutamine (Q), asparagine (N), leucine (L), phenylalanine (F), isoleucine (I), methionine (M), valine (V), alanine (A), cysteine (C), or tyrosine (Y) in their peptide sequences [6,82,83].

Plant-based antioxidant compounds counter-react with the reactive oxygen and nitrogen (ROS/RON) species and activate signal cascade systems inside the cells. The chain reactions of ROS/RON can take place with either hydrogen atom transfer (HAT) or single-electron transfer via proton transfer (SET-PT), or sequential electron transfer via proton transfer or transition metal chelation (TMC). Accordingly, these compounds stabilize the free radicals produced during the breakdown of lipids [4,82,120,121].

The stability of the animal-derived antioxidants depends on the temperature, pH, concentration of salts, and the enzymes used for hydrolysis. Since these peptides are small in molecular weight and stable structure, the activity and absorbance into the bloodstream are very high. These antioxidant peptides act on the lipid oxidation promoters, such as Fe^2+^, Cu^2+^, H_2_O_2_, lipid peroxides, NO, and other aldehydes, to prevent or delay lipid oxidation in food systems, which will prevent the damaging of vital compounds in the cells, such as DNA, proteins, lipids, and hormones. However, the ability of antioxidant peptides to prevent an oxidative reaction is not clear yet [120,122].

## 5. Applications of Antioxidants

Bioactive peptides are used as therapeutic agents to improve human health. In addition to their antioxidant activity, peptides can act as antimicrobial, anti-inflammatory, anticancer, or immunomodulatory agents and prevent type II diabetes. Many researchers suggested the potential of using antioxidant peptides as a natural antioxidant in food products while improving their sensory properties [120]. Plant-derived antioxidants are used extensively in the food industry to improve the foods’ shelf life, color, texture, and sensory properties. The production of plant-based antioxidants is simple and cheap, and their antioxidant effects have a stronger antioxidant capacity than the animal-derived ones [25,72]. These natural antioxidants derived from plant and animal sources are recommended as replacers for the synthetic antioxidants used in the food industry because natural antioxidants are considered safe and have fewer adverse health effects.

Compared with animal-based antioxidants, plant-based antioxidants are more practical for use in food processing because they are highly effective at low concentrations and a variety of antioxidant compounds can be extracted from a single plant source. Although some animal-derived antioxidants are known to have a comparable antioxidant capacity to synthetic ones, their production cost for separating specific antioxidant peptides is too expensive for practical use. Therefore, if animal- or plant-based antioxidants are used only to prevent lipid oxidation in foods, plant-based antioxidants are better. However, if the whole enzyme hydrolysate is used to produce animal-based antioxidants used in food, various peptides, including the specific antioxidant peptides, can provide many other biological properties that can benefit human health. Therefore, instead of separating and using a specific antioxidant peptide, whole protein hydrolysate is better to use as an ingredient in a food product. This concept is important because people are more concerned about their health, and including ingredients with natural antioxidants and functional peptides in their food products will satisfy consumers’ needs.

## 6. Conclusions

As a concluding remark, the authors would like to emphasize that plant-based antioxidants are a good source of antioxidants that can be used as food preservative agents. However, animal-based antioxidants (individual or multiple peptides) should not be considered as antioxidants or recommended as antioxidant agents in foods. Rather, they should be considered an ingredient to fulfill the nutritional requirements of the food with some antioxidant activities.

## Figures and Tables

**Table 1:** Most common plant-based phenolic antioxidants and their potential applications.

Antioxidant Type	Subgroups	Examples	Applications	References
Phenolic compounds	Phenolic acids	Salicylic acid, Gentisic acid, *p*-Hydoxybenzoic acid, Protocatechuic acid, Vanillic acid, Syringic acid, Gallic acid, *p*-coumaric acid, Ferulic acid, Caffeic acid, Sinapic acid	As naturally present, primary antioxidant.	[21,25,35]
	Stilbenes	Piceid, Resveratrol, Piceatannol, Pterostilbene	Antioxidant activity against proteins and lipids	[27,28]
	Tannins	Biopolymers based on flavan-3-ols and Gallic and Ellagic acid	Strong antioxidant activities than flavonoids and phenolic acids	[36,37,38,39,40,41]
	Flavonoids	Flavone, Flavanol, Flavanone, Flavanonol, Flavonone, Flanononol, Flavanol (catechin), Isoflavone, Anthocyanidin	Act as antioxidant compounds if fruits, berries	[24,26,42,43,44]
	Lignans	Secoisolariciresinol, Matairesinol, Pinoresinol, Lariciresinol, sesamin, sesamolin	Strong antioxidant activities	[30,32]

**Table 2:** Most common animal-based antioxidant proteins and peptides.

Origin	Example Bioactive Proteins/Peptides		References
Milk, milk-based products, and milk by-products	VLPVPQK, RLDGQGRPRVWLGR, KVLPVPEK, TPDNIDIWLGGIAEPQVKR, AVPYPQR, NPYVPR, ARHPHPHLSFM, VAYSDDGENWTEYRDQGAVEGK, YFYPEL		[85,92,93,95,97,98]
Meat and meat products	Naturally present peptides	Carnosine (β-alanyl-*_L_*-histidine), Anserine (*N*-β-alanyl-1-methyl-*_L_*-histidine), Glutathione (GSH, Glu-Cys-Gly)	[106,117,118]
	Synthetic Peptides	DAQEKLE, AKHPSDFGADAQ, SLTA, VT, AKHPSDFGADAQA, KAPVA, PTPVT, RPR, GLSDGEWQ, GFHI, DFHING, FHG, DSGVT, IEAEGE, EELDNALN, VPSIDDQEELM, DAQEKLE, DSGVT, IEAEGE, EELDNALN, VPSIDDQEELM, DAQEKLE, ATA, SLTA, VT, SAGNPN. GLAGA, DLEE, FGG, DM, ALTA	[3,85,87,88,91,99,100,101,104,105,106,107,108]
Slaughterhouse by-products	QGAR, LQGM, LQGMH, HC, QYDQGV, YEDCTDCHN, AADNANELFPPN, GAHTHPRLPFKPR, GMPGFDVR, ADASVLPK, GAHQPSG, QQPVRDOQ		[84,86,88,101,109,110,111,113,114]
Chicken eggs	Proteins	Ovotransferrin, Phosvitin	[79,80]
	Peptides	White–WNIP, GWNI, IRW, LKP, VYLPR, YLGAK, GGLEPINFN, ESKPV, DVYSF, DSTRTQ Yolk–LMSYMWSTSM, KLSDW, RASDPLLSV, QSLVSVPGMS, LELHKLRSSHWFSRR, YINQMPQKSRE, RNDDLNYIQ, YINQMPQKSREA, VTGRFAGHPAAQ, LAPSLPGKPKPD, KGLWE, AGTTCLFTPLALPYDYSH, YIEAVNKVSPRAGQF	[78,79,80,91,115,116]
